# Factors related to health-related quality of life in ankylosing spondylitis, overall and stratified by sex

**DOI:** 10.1186/s13075-018-1784-8

**Published:** 2018-12-27

**Authors:** Lucy Law, Jeanette Beckman Rehnman, Anna Deminger, Eva Klingberg, Lennart T. H. Jacobsson, Helena Forsblad-d’Elia

**Affiliations:** 1 Department of Public Health and Clinical Medicine, Rheumatology, Umeå University, 901 87 Umeå, Sweden; 2 Department of Rheumatology and Inflammation Research, Sahlgrenska Academy at University of Gothenburg, Box 480, 405 30 Gothenburg, Sweden

**Keywords:** Ankylosing spondylitis, Health-related quality of life, Medical outcome survey short form-36, Disease activity, Fatigue, Cross-sectional study, Observational study

## Abstract

**Background:**

Ankylosing spondylitis (AS) begins early in life and often leads to reduced physical function, but less is known about the impacts it has on health-related quality of life (HRQoL). The aims of this study were to assess HRQoL using the Short Form-36 (SF-36) in a cohort of patients with AS compared with controls and to examine associations between SF-36 scores and spinal radiographic changes, physical function, disease activity and demographic data overall and stratified by sex.

**Methods:**

A cohort of patients with AS from Western Sweden were assessed using the Modified Stoke Ankylosing Spondylitis Spine Score (mSASSS) with spinal radiographs, clinical examination and questionnaires, including the Bath Ankylosing Spondylitis Metrology Index, Bath Ankylosing Spondylitis Functional Index (BASFI), Ankylosing Spondylitis Disease Activity Score-C-reactive protein (ASDAS-CRP), Bath Ankylosing Spondylitis Disease Activity Index, Bath Ankylosing Spondylitis Patient Global (BASG) and SF-36. Each patient’s SF-36 results were compared with those of five age-matched and sex-matched persons (*n* = 1055) from the SF-36 Swedish normative population database. Associations between SF-36 physical component summary (PCS) and mental component summary (MCS) scores and disease-related and demographic factors were investigated using univariate and multivariable ogistic regression analyses with PCS and MCS below/above their respective median values as dependent variables.

**Results:**

A total of 210 patients, age (median, IQR) 49.0 (21.2) years, symptom duration 24.0 (21.0) years, men 57.6% and HLAB27 87.1% were included. Patients with AS scored significantly lower (*p* < 0.001) compared to controls in all SF-36 domains and component summaries; PCS 42.4 (14.5) in AS versus 52.4 (11.8) in controls and MCS 47.9 (20.0) in AS versus 54.1 (10.1) in controls. Both men and women scored significantly lower in PCS compared with MCS. Multivariable logistic regression analyses revealed that living without a partner (OR 2.38, 95% CI 1.00–5.67), long symptom duration (year in decade OR 1.66, 95% CI 1.16–2.37), higher BASFI (OR 1.98, 95% CI 1.46–2.70) and ASDAS ≥ 2.1 (OR 3.32, 95% CI 1.45–7.62) were associated with worse PCS, while living without a partner (OR 3.04, 95% CI 1.34–6.91), fatigue (visual analogue scale for global fatigue greater than the median (OR 6.36, 95% CI 3.06–13.19) and ASDAS ≥ 2.1 (OR 2.97, 95% CI 1.41–6.25) with worse MCS. Some differences between sexes were observed in the results.

**Conclusions:**

The patients with AS had significantly lower HRQoL compared with controls. PCS was more affected compared to MCS in both sexes. Both disease-related and demographic factors were associated with HRQoL, partly overlapping for PCS and MCS. Factors associated with HRQoL showed some differences between sexes. By modifying factors, such as ASDAS-CRP and fatigue, HRQoL may potentially be improved.

**Trial registration:**

ClinicalTrials.gov, NCT00858819. Registered on 9 March 2009. Last updated on 28 May 2015.

**Electronic supplementary material:**

The online version of this article (10.1186/s13075-018-1784-8) contains supplementary material, which is available to authorized users.

## Background

Ankylosing spondylitis (AS) is a chronic inflammatory rheumatic disease primarily affecting the sacroiliac joints and spine [1]. Inflammation of the spinal structures and progressive spinal changes in the vertebrae and surrounding tissue, is largely responsible for the decreased physical function and mobility experienced by patients with AS [2]. Studies of AS often describe functional disabilities and measures of disease activity, however, they less often report the quality of life experienced by patients with AS and how this is related to AS disease characteristics. Health-related quality of life (HRQoL) is a multi-dimensional concept including not only a person’s physical wellbeing, but also a person’s mental health and physical ability, both as an individual and as a participating member of the community. The Medical Outcome Survey Short Form-36 (SF-36) was designed for use in clinical practice and research, health policy evaluations, and general population surveys and is utilised in many different countries [3–6]. Yang et al. recently performed a meta-analysis based on of 38 studies assessing HRQoL using the SF-36 in patients with AS and found that they had significantly worse HRQoL compared to persons from general populations and that to measure HRQoL should be regarded as an essential part of the overall assessment of patients with AS [7]. The SF-36 is grouped into eight domains reflecting physical and mental health and two summary scores, a physical component summary (PCS) and a mental component summary (MCS) score. Yang et al. reported that pooled mean scores ranged from 45.9 to 58.2 in the physical health domains and from 47.5 to 62.5 in the mental health domains in their meta-analysis of patients with AS [7] as compared to 72.5 to 85.2 and 60.0 to 84.6, respectively, in the normative general population database from Norway, one of the databases used for comparison with AS [8]. The Bath Ankylosing Spondylitis Disease Activity Index (BASDAI) and Bath Ankylosing Spondylitis Functional Index (BASFI) have been found to be negatively associated with some domains of the SF-36 [7, 9–13]. The relationship between HRQoL, laboratory markers of inflammation, the Ankylosing Spondylitis Disease Activity Score (ASDAS), Bath Ankylosing Spondylitis Metrology Index (BASMI) and spinal radiographic changes, as assessed by the modified Stoke Ankylosing Spondylitis Spinal Score (mSASSS), is less studied. Since the AS phenotype differs between sexes, with men generally displaying more AS spinal radiographic changes compared to women, it is of importance to investigate factors related to HRQoL overall and also stratified by sex to be able to better personalize the care of patients with AS [14, 15].

The objectives of this study were to investigate the following in patients with AS: (1) HRQoL assessed by the SF-36 compared with HRQoL in controls from the general population, (2) HRQoL in relation to age and (3) to examine associations between the SF-36 scores and spinal radiographic changes, physical function, mobility, disease activity and demographic data overall and stratified by sex.

## Methods

### Patients and controls

Patients were recruited from three sites in Western Sweden [14, 16]. This cross-sectional study focuses on HRQoL assessed by the SF-36 questionnaire. The inclusion criteria for the study were a diagnosis of AS according to the modified New York criteria [17] and age ≥ 18 years. The exclusion criteria were difficulties in understanding the Swedish language, dementia and pregnancy. Patients with psoriasis or inflammatory bowel disease (IBD) were also excluded in order to create a more homogenous cohort of patients with typical AS. All patients with AS meeting the study criteria were invited to participate and of these, 211 patients were initially included.

**Fig. 1:**
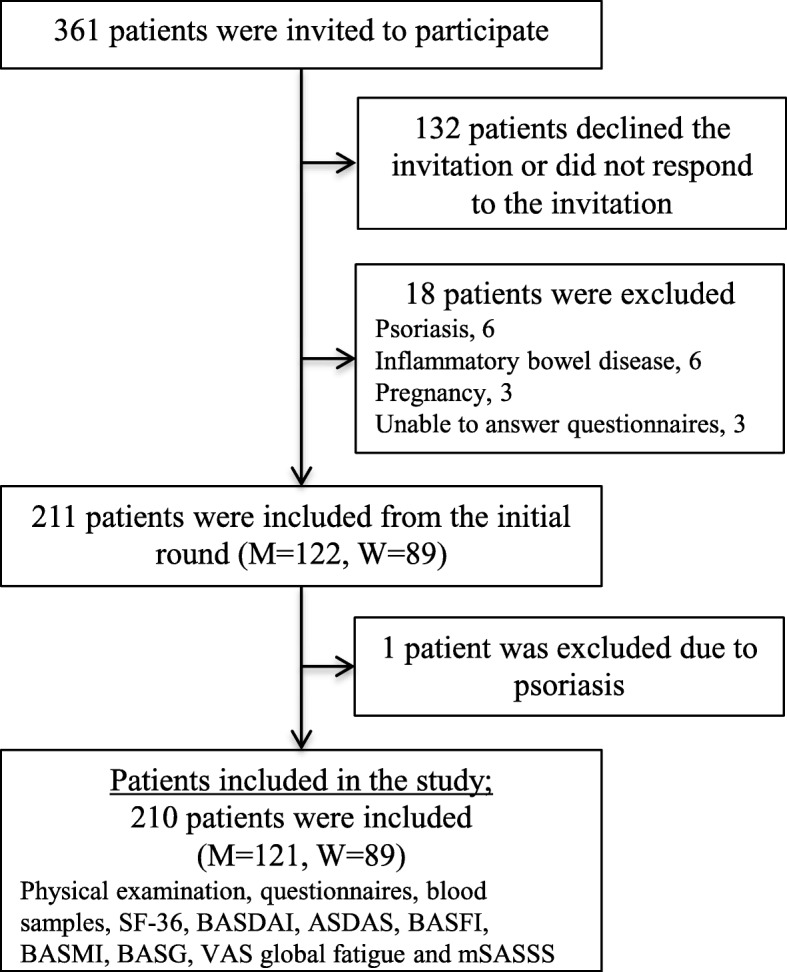
Process of inclusion of the patients with ankylosing spondylitis. M, men; W, women; BASDAI, Bath Ankylosing Spondylitis Disease Activity Index; ASDAS, Ankylosing Spondylitis Disease Activity Score; BASFI, Bath Ankylosing Spondylitis Functional Index; BASMI, Bath Ankylosing Spondylitis Metrology Index; BAS-G, Bath Ankylosing Spondylitis Patient Global; VAS, visual analogue scale; mSASSS, modified Stoke Ankylosing Spondylitis Spinal Score

Results of the SF-36 questionnaires completed by the patients with AS (*n* = 211) were compared to those of five age-matched and sex-matched persons, per AS patient (*n* = 1055), randomly drawn from the SF-36 Swedish normative population database [5]. One of the included patients was later found to have psoriasis and was excluded from further analysis, leaving 210 patients to take part in this study. Figure 1 summarises the process of patient inclusion.

This study was approved by the Regional ethical committee at the University of Gothenburg, Sweden and was performed in accordance with the Declaration of Helsinki.

### The SF-36 questionnaire

HRQoL was assessed using the self-reported SF-36 questionnaire, an instrument consisting of 36 questions grouped into eight domains [3–6]. The eight domains include physical functioning (PF; 10 items), role physical (RP; 4 items), bodily pain (BP; 2 items), general health (GH; 5 items), vitality (VT; 4 items), social functioning (SF; 2 items), role emotional (RE; 3 items) and mental health (MH; 5 items). In addition, a further single item concerns reported health transition over the past year. The response alternatives for the role limitation domains (physical and emotional) are dichotomous (yes/no), and the other items have three to six response choices. The items within each domain contribute to an overall domain score between 0 and 100, with 0 representing worst and 100 excellent health. The questions reflect the individuals’ health status during the last 4 weeks. The eight domains are also combined to produce two summary scores. The first four aforementioned domains, PF, RP, BP and GH, give an overall physical component summary (PCS) score and the second four domains, VT, SF, RE and MH, give an overall mental component summary (MCS) score. The component summary scores are standardised combined scores, with a mean of 50 and standard deviation of 10.

### Other questionnaires

Participants also answered additional questionnaires including the BASDAI and the BASFI. The BASDAI estimates the patient’s disease activity and the BASFI estimates the functional limitations during the last week. Higher values indicate higher disease activity and respectively worse physical function [18].

The Ankylosing Spondylitis Disease Activity Score, including C-reactive protein (ASDAS-CRP), was calculated for each patient. The disease activity is divided into inactive disease (ASDAS < 1.3), low disease activity (1.3 ≤ ASDAS < 2.1), high disease activity (2.1 ≤ ASDAS ≤ 3.5) and very high disease activity (ASDAS > 3.5) [18, 19]. The patients also answered the Bath Ankylosing Spondylitis Patient Global (BAS-G) [18]. A visual analogue scale (VAS) for global fatigue was used to evaluate fatigue experienced over the last few days graded from not at all (0) to extreme fatigue (100). In addition, the patients answered a questionnaire about lifestyle habits, medical history and medication.

### Physical examination

The patients’ mobility was measured using the BASMI. A higher BASMI score indicates less spinal mobility [18]. Physical examinations were performed by one physician (EK).

### Radiography

Spinal radiographic changes were assessed from the lateral projection of the spinal radiographs and were graded using the mSASSS. Anterior corners of vertebra C2–T1 and T12–S1 were graded with a score between 0 and 3 (0 = normal, 1 = erosion, sclerosis or squaring, 2 = syndesmophyte, 3 = bridging syndesmophyte). The overall scoring scale ranges from 0 to 72, with 72 representing complete ankylosis [20]. To have a mSASSS ≥ 2 at a vertebral corner was classified as having a syndesmophyte. Severe spinal radiographic change was defined as ≥ 3 consecutive intervertebral bridges in the cervical spine and/or the lumbar spine, similar to the definition of grade 4 = severe in the Bath Ankylosing Spondylitis Radiology Index (BASRI) [21]. The majority of x-rays were performed within a couple of months after the study inclusion and < 5% of the patients were examined up to 6 months after the inclusion. One experienced radiologist scored all radiographs.

### Laboratory tests

Erythrocyte sedimentation rate (ESR) and C-reactive protein (CRP) were analysed consecutively by standard laboratory techniques at the different hospitals.

### Statistics

Non-parametrical statistical tests were applied because not all data were normally distributed. The median, 25th percentile (Q1) and 75th percentile (Q3) or interquartile range (IQR) or mean with standard deviation (SD) were presented for continuous data and frequency with percentage for categorical data. Data were compared using the Mann–Whitney U test or chi square test, as appropriate. The Kruskal–Wallis test was used to compare multiple groups and the Mann–Whitney U test was applied for post hoc analyses with a Bonferroni-corrected *p* value (<0.008). Univariate logistic regression analyses were conducted with dichotomised PCS and MCS below (coded 1) and above (coded 0) their respective median values as dependent variables and demographic and disease-related variables of interest as covariates. Age and symptom duration were stratified into decades and BASMI and BASFI into whole units. BASDAI was dichotomised into ≥ 4 (coded 1) and < 4 (coded 0) and ASDAS-CRP into ≥ 2.1 (coded 1) and < 2.1 (coded 0). mSASSS+ 1 was log-transformed in order to improve the distribution. Variables with *p* values ≤0.2 in the univariate analyses (Additional file 1: Tables S4 and S5) were chosen first to potentially be entered into the multivariable logistic regression models, the enter method. Thereafter, variables were excluded if the variable of interest by definition was a part of another variable, for instance, ASDAS-CRP was chosen as a variable reflecting disease activity instead of the BASDAI and CRP. The BASG was excluded as the ASDAS-CRP was chosen. The VAS for global fatigue was chosen instead of the BASDAI. The remaining variables were thereafter checked for multicollinearity and variables with the lowest *p* value in the univariate analysis were kept and entered into the multivariable models. In the multivariable logistic regression analyses, PCS and MCS were dichotomised as described above and were the dependent variables, while the other variables formed the independent variables (Tables 2 and 3). A *p* value <0.05 was considered statistically significant. All statistical analyses were performed using SPSS version 24 (SPSS Inc., IBM, Chicago, USA).

## Results

For the enrolment process see Fig. 1. A total of 210 patients with AS, 121 (57.6%) men and 89 (42.4%) women, with a median age of 49.0 years (40.0, 61.2) and a median symptom duration of 24.0 years (13.0, 34.0), were included in this report. HLA-B27 was present in 183 (87.1%) of the patients with AS. The mSASSS median and mean values were 5.0 (0.0, 20.0) and 13.9 ± 19.0, respectively. Concerning biological drugs, 43 (20.5%) patients were treated with a TNF-inhibitor (TNFi) out of which 32 (15.2%) were used in combination with a conventional synthetic disease modifying anti-rheumatic drug (csDMARD). In total, 73 (34.8%) patients were on a TNFi and/or a csDMARD. Characteristics of all the patients and stratified by sex are displayed in Table 1. Comparisons between men and women showed that the age and symptom duration did not differ significantly between sexes. Women with AS had a significantly higher ESR and VAS for global fatigue compared to the men. Men with AS had significantly higher mSASSS and body mass index (BMI) than the women. More men carried HLA-B27 and had ≥ 1 prevalent syndesmophyte compared to the women. Only men had severe radiographic changes in the spine, defined as ≥ 3 consecutive intervertebral bridges in the cervical spine and/or the lumbar spine. Otherwise, baseline characteristics were similar between sexes.

**Table 1:** Characteristics of the patients with ankylosing spondylitis, overall and stratified and compared by sex

Variables	All, *n* = 210	Men, *n* = 121 (57.6)	Women, *n* = 89 (42.4)	*P* value
Age, years	49.0 (40.0, 61.2)	49.0 (39.5, 61.0)	49.0 (41.5, 62.0)	0.48
BMI, kg/m^2^	25.2 (22.8, 28.4)	25.8 (23.6, 28.9)	24.0 (21.8, 27.5)	**0.003**
Civil state: single	58 (27.6)	34 (28.1)	24 (27.0)	0.86
in a relationship	152 (72.4)	87 (71.9)	65 (73.0)	0.87
Years in school: < 13	98 (46.9)^a^	62 (51.7)^a^	36 (40.4)	0.11
≥ 13 years	111 (53.1)	58 (48.3)	53 (59.6)	0.11
Ever smoker	105 (50.0)	65 (53.7)	40 (44.9)	0.21
VAS global fatigue, score	59.0 (27.0, 74.0)	50.0 (20.5, 68.0)	64.0 (42.5, 76.0)	**0.002**
Duration of symptoms, years	24.0 (13.0, 34.0)^c^	23.0 (13.0, 33.0)^b^	24.0 (12.5, 34.0)^a^	0.80
Duration of disease, years	12.0 (5.0, 23.0)^a^	12.5 (6.0, 24.0)^a^	11.0 (4.0, 18.5)	0.11
ESR, mm/h	11.0 (7.0, 19.0)	10.0 (5.0, 17.0)	14.0 (10.0, 22.5)	**0.001**
CRP, mg/L	3.0 (1.0, 7.0)^a^	3.0 (1.0, 7.0)^a^	2.0 (1.0, 7.5)	0.38
HLA-B27 positive	183 (87.1)	112 (92.6)	71 (79.8)	**0.006**
History of anterior uveitis	108 (51.5)	69 (57.0)	39 (43.8)	0.058
TNFi and/or csDMARD	73 (34.8)	45 (37.2)	28 (31.5)	0.39
BASMI, score	3.0 (2.0, 4.0)	3.0 (1.8, 4.3)	2.8 (2.2, 3.8)	0.34
BASFI, score	2.3 (1.0, 3.8)^c^	2.3 (1.0, 3.7)^a^	2.3 (1.0, 4.2)^b^	0.73
BASDAI, score	3.5 (1.7, 5.3)^d^	2.9 (1.5, 5.2)^c^	3.7 (1.9, 5.6)^a^	0.079
ASDAS-CRP, score	2.1 (1.4, 2.9)^a^	2.0 (1.3, 2.9)^a^	2.2 (1.5, 2.8)	0.70
BAS-G, score	2.90 (1.15, 5.45)	2.6 (1.0, 5.0)	3.5 (1.3, 5.7)	0.28
mSASSS, score	5.0 (0.0, 20.0)	8.0 (2.0, 34.0)	2.0 (0.0, 9.5)	**< 0.001**
Syndesmophyte and/or	98 (46.7)	71 (58.7)	27 (30.3)	**< 0.001**
≥ 3 intervertebral bridges consecutively, cervical and/or lumbar spine	29 (13.8)	29 (24)	0	**< 0.001**
PF, score	80.0 (65.0, 90.0)	80.0 (69.4, 90.0)	75.0 (60.6, 90.0)	**0.023**
	74.4 ± 21.4	76.7 ± 21.5	71.2 ± 21.0	
RP, score	75.0 (25.0, 100.0)	75.0 (25.0, 100.0)	75.0 (0.0, 100.0)	0.12
	59.3 ± 41.1	63.1 ± 40.3	54.2 ± 41.8	
BP, score	51.0 (41.0, 64.0)	51.0 (41.0, 72.0)	51.0 (41.0, 62.0)	0.19
	54.1 ± 21.9	55.8 ± 22.8	51.7 ± 20.4	
GH, score	62.0 (40.0, 77.0)	62.0 (40.0, 77.0)	60.0 (40.0, 76.0)	0.78
	57.1 ± 22.8	56.6 ± 23.7	57.7 ± 21.8	
VT, score	50.0 (30.0, 70.0)	55.0 (30.0, 75.0)	50.0 (30.0, 65.0)	**0.010**
	50.6 ± 23.6	54.3 ± 24.1	45.5 ± 22.0	
SF, score	75.0 (65.2, 100.0)	87.5 (62.5, 100.0)	75.0 (62.5, 100.0)	0.10
	75.8 ± 25.0	77.7 ± 25.6	73.3 ± 24.1	
RE, score	100.0 (33.3, 100.0)^b^	100.0 (33.3, 100.0)^a^	100.0 (33.3, 100.0)^a^	0.16
	68.8 ± 40.8^b^	71.9 ± 39.8^a^	64.4 ± 41.9^a^	
MH, score	76.0 (63.0, 88.0)	76.0 (64.0, 88.0)	72.0 (60.0, 84.0)	**0.039**
	71.9 ± 19.8	73.7 ± 20.7	69.5 ± 18.3	
PCS, score	42.4 (34.3, 48.7)^b^	43.6 (35.4, 49,4)^a^	41.8 (32.6, 47.5)^a^	0.16
	41.4 ± 10.0^b^	42.1 ± 10.0^a^	40.4 ± 10.2^a^	
MCS, score	48.0 (35.6, 55.2)^b^	49.2 (39.9, 56.1)^a^	46.0 (33.4, 53.8)^a^	0.13
	44.8 ± 12.5^b^	45.9 ± 12.3^a^	43.3 ± 12.8^a^	

Values are median and 25th percentile (Q1) and 75th percentile (Q3), numbers of patients and percent (%) or mean ± SD. Comparisons between men and women are assessed by Mann-Whitney U-test or Chi-square test. Significant differences are in bold text

*BMI* body mass index, *VAS* visual analogue scale, *ESR* erythrocyte sedimentation rate, *CRP* C-reactive protein, *TNFi* TNF inhibitor, *csDMARD* conventional synthetic disease modifying anti-rheumatic drug, *BASMI* Bath Ankylosing Spondylitis Metrology Index, *BASFI* Bath Ankylosing Spondylitis Functional Index, *BASDAI* Bath Ankylosing Spondylitis Disease Activity Index, *ASDAS-CRP* Ankylosing Spondylitis Disease Activity Score-CRP, *BAS-G* Bath Ankylosing Spondylitis Patient Global, *mSASSS* Modified Stoke Ankylosing Spondylitis Spine Score, *PF* physical function, *RP* role physical, *BP* bodily pain, *GH* general health, *VT* vitality, *SF* social function, *RE* role emotional, *MH* mental health, *PCS* physical component summary, *MCS* mental component summary

Number of missing data values: ^a^*n* = 1; ^b^*n* = 2;^c^*n* = 3; ^d^*n* = 4

### Health related quality of life in patients with ankylosing spondylitis and in the general population

**Fig. 2:**
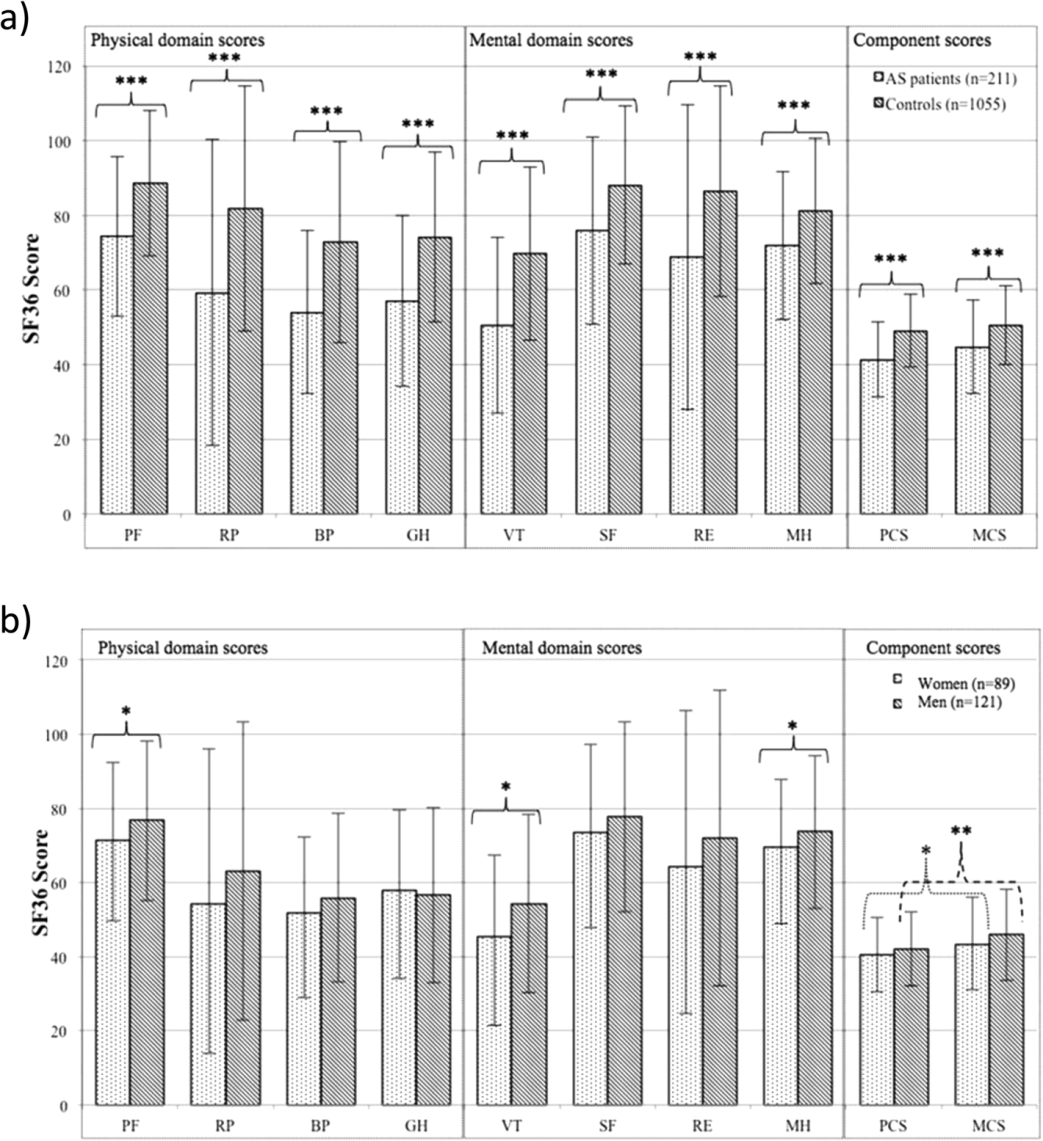
Short form-36 (SF-36) in 211 patients with ankylosing spondylitis (AS) compared to 1055 age-matched and sex-matched controls from the general population (**a**) and 210 patients with AS, stratified by sex (**b**). Mean ± SD are shown. physical component summary (PCS) and mental component summary (MCS) are standardized scores with a mean of 50 and SD of 10. The other domains score between 0 and 100 with 0 = worst and 100 = best health. PF, physical function; RP, role physical; BP, bodily pain; GH, general health; VT, vitality; SF, social function; RE, role emotional; MH, mental health. Mann-Whitney U test and Wilcoxon rank sum tests were used for comparisons; *p < 0.05, **p < 0.01, ***p < 0.001

The patients with AS had significantly lower SF-36 scores in all domains and the component summary scores, PCS and MCS, when compared to five age-matched and sex-matched controls from the general population per AS patient (*p* < 0.001) (Fig. 2a, Additional file 1: Table S1). The mean PCS score ± SD was 41.3 ± 10.1 in AS versus 49.1 ± 9.8 in controls and the mean MCS score was 44.6 ± 12.7 in AS versus 50.5 ± 10.4 in controls. The mean age ± SD was 50.1 ± 12.9 years in the patients with AS versus 50.1 ± 12.8 years in the controls, and the sex-distribution was exactly the same, due to the matched selection of controls.

Comparison of SF-36 physical domain scores between sexes in AS showed that women had a significantly lower PF score compared to men (*p* = 0.023). Regarding the mental domains, women had significantly lower VT and MH scores compared to men (*p* = 0.010 and *p* = 0.039, respectively) (Fig. 2b, Table 1). No significant differences were found in PCS or MCS between men and women with AS. PCS was significantly lower compared with MCS in the total AS group (*p* < 0.001), a difference also seen in both women and men in the sex-stratified analyses (*p* = 0.027 and *p* = 0.002 respectively) (Fig. 2b).

### Health-related quality of life in relation to age

**Fig. 3:**
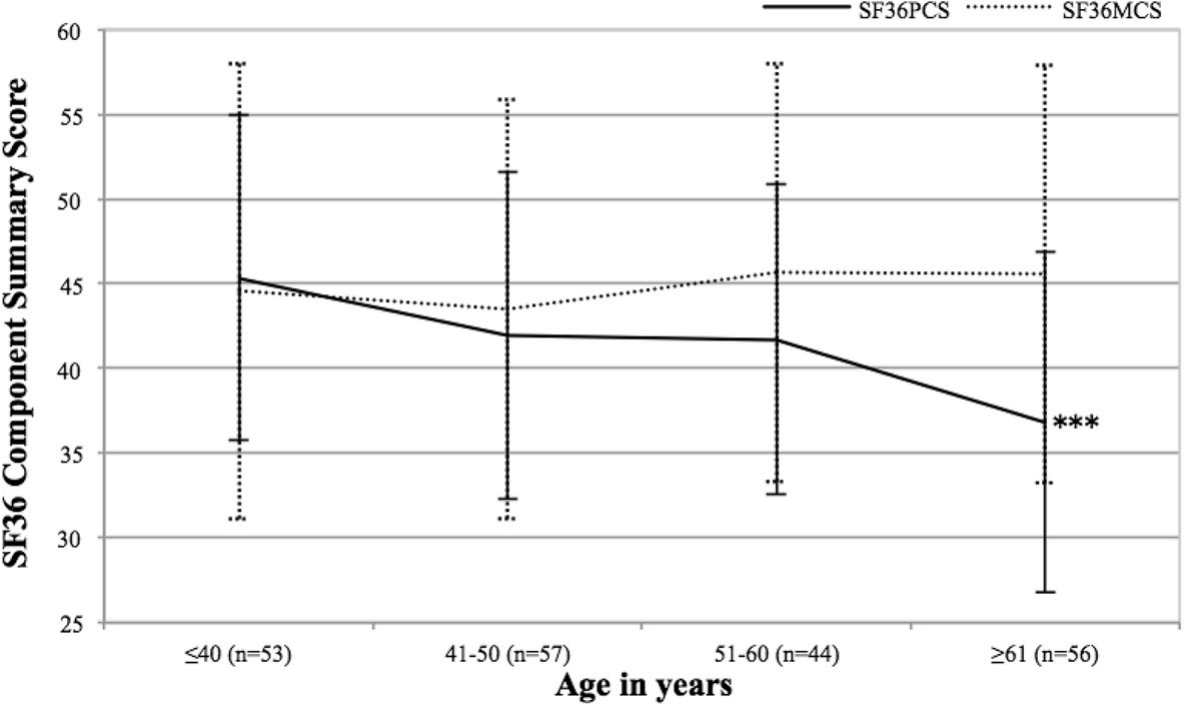
Patients with ankylosing spondylitis (*n* = 210) divided in four age groups displaying Short Form-36 (SF-36) physical component summary (PCS) and mental component summary (MCS) scores. Mean ± SD are shown; ***p < 0.001, n, number of patients

The patients were divided into four different age groups: ≤ 40 years (*n* = 53), 41–50 years (*n* = 57), 51–60 years (*n* = 44) and ≥ 61 years (*n* = 56), in order to study the impact of age on the SF-36 summary scores. There was a difference in the PCS score between the four age groups (*p* < 0.001), and post hoc analysis showed that the PCS score was significantly lower in the age group ≥ 61 years compared to the age group ≤ 40 years (*p* < 0.001). No difference in the MCS score between the 4 age groups was found, Fig. 3. Correlation analyses revealed an inverse association between PCS score and age (*r*_s_ = − 0.29, *p* < 0.001), and when stratified by sex, there was significant association in men (*r*_s_ = − 0.34, *p* < 0.001) and a trend in women (*r*_s_ = − 0.20, *p* = 0.066). There was no significant correlation between MCS and age.

### Comparison between patients with ankylosing spondylitis with higher and lower health-related quality of life scores

The patients were divided into groups according to the median values of PCS and MCS scores and different characteristics were compared between patients with lower versus higher PCS and MCS scores. The comparisons were also performed stratified by sex. The results are shown in Additional file 1: Tables S2 and S3.

### Logistic regression analyses demonstrating factors associated with worse health-related quality of life

Univariate logistic regression analyses with PCS and MCS, below their respective median values, as dependent variables and demographic and disease-related factors as independent variables, overall and stratified by sex, are shown in Additional file 1: Tables S4 and S5.

The process for including variables into the multivariable regression analyses is described in “Methods”. Multivariable logistic regression analyses showed that to live alone without a partner was a significant factor associated with a worse PCS score (OR 2.38, 95% CI 1.00–5.67). To have a longer symptom duration (decades) (OR 1.66, 95% CI 1.16–2.37), higher BASFI (OR 1.98 per unit, 95% CI 1.46–2.70) and ASDAS-CRP ≥ 2.1 (OR 3.32, 95% CI 1.45–7.62) was also associated with a worse PCS score. The sex-stratified analyses revealed that, in men, the most important determinants for a worse PCS score were a high BASFI and an ASDAS-CRP ≥ 2.1, compared to a high BASFI in women (Table 2). In men, to have severe spinal radiographic changes was associated with a worse PCS score in the univariate but not in the multivariable logistic regression analysis. In a sensitivity analysis, age in decades was inserted into the models instead of symptom duration in decades. Age in decades was not a significant factor in these models, thus symptom duration in decades was kept in the models according to the criteria described in “Methods”.

**Table 2:** Multivariable logistic regression analyses with PCS score below the median value as dependent variable

	All, *n* = 210 PCS < median PCS 42.4			Men, *n* = 121 PCS < median PCS 43.6			Women, *n* = 89 PCS < median PCS 41.8		
Variables	OR	95% CI	*P* value	OR	95% CI	*P* value	OR	95% CI	*P* value
BMI, kg/m^2^	1.03	0.93 to 1.14	0.55	0.98	0.84 to 1.14	0.78			
Civil state, single	2.38	1.00 to 5.67	**0.049**	3.28	0.94 to 11.52	0.063	3.07	0.83 to 11.40	0.094
Years in school, < 13 years	1.12	0.50 to 2.48	0.79	1.40	0.43 to 4.55	0.57			
Ever smoker	0.77	0.35 to 1.73	0.53	0.55	0.16 to 1.89	0.34			
VAS global fatigue, > median	1.04	0.46 to 2.38	0.92	2.23	0.66 to 7.56	0.20			
Duration of symptoms, decades	1.66	1.16 to 2.37	**0.005**	1.36	0.81 to 2.30	0.24	1.69	0.99 to 2.90	0.055
ESR, > median	0.98	0.45 to 2.10	0.95						
BASMI, units							0.72	0.38 to 1.34	0.30
Lateral spinal flexion, cm	0.99	0.90 to 1.09	0.86	0.92	0.80 to 1.06	0.27			
BASFI, unit	1.98	1.46 to 2.70	**< 0.001**	2.09	1.29 to 3.41	**0.003**	2.25	1.42 to 3.57	**0.001**
ASDAS-CRP, ≥ 2.1	3.32	1.45 to 7.62	**0.005**	4.87	1.46 to 16.27	**0.010**	1.83	0.51 to 6.53	0.36
Log10mSASSS + 1, score	0.74	0.36 to 1.54	0.43						
≥ 3 intervertebral bridges consecutively (only men)^a^				0.57	0.12 to 2.70	0.48			

Significant differences are in bold text. The independent variables were defined by having a univariate association with PCS (p-value ≤0.2) and not demonstrating collinearity. Due to some missing data, the models are based on an overall 203 patients and in stratified analyses 115 men and 86 women

*PCS* physical component summary, *OR* odds ratio, *CI* confidence interval, *BMI* body mass index, *VAS* visual analogue scale, *ESR* erythrocyte sedimentation rate, *BASMI* Bath Ankylosing Spondylitis Metrology Index, *BASFI* Bath Ankylosing Spondylitis Functional Index, *ASDAS-CRP* Ankylosing Spondylitis Disease Activity Score C-reactive protein, *mSASSS* Modified Stoke Ankylosing Spondylitis Spine Score ^a^Cervical and/or lumbar spine

To live alone (OR 3.04, 95% CI 1.34–6.91), to have VAS global fatigue greater than the median value (OR 6.36, 95% CI 3.06–13.19) and ASDAS-CRP ≥ 2.1 (OR 2.97, 95% CI 1.41–6.25), were significant determinants associated with a worse MCS score (Table 3). The sex-stratified analyses revealed that longer education, high fatigue, and no severe radiographic changes in men, and to live without a partner, have high fatigue and ASDAS-CRP ≥ 2.1 in women were the most important determinants of a worse MCS score (Table 3). In sensitivity analysis, age in decades was inserted into the models but was not a significant factor and did not alter the models substantially and was therefore not used. There was no significant association between the mSASSS and the PCS or MCS in the univariate or multivariable logistic regression analyses. Further sensitivity analyses based on demographics, excluding disease-related variables, revealed that to live without a partner was still associated with both worse PCS and MCS scores, and age in decades was associated with a worse PCS score but not with a worse MCS score (Additional file 1: Table S6).

**Table 3:** Multivariable logistic regression analyses with MCS score below the median value as dependent variable

	All, *n* = 210 MCS < median MCS 48.0			Men, *n* = 121 MCS < median MCS 49.2			Women, *n* = 89 MCS < median MCS 46.0		
Variables	OR	95% CI	*P* value	OR	95% CI	-value	OR	95% CI	*P* value
BMI, kg/m^2^	1.01	0.93 to 1.09	0.86						
Civil state, single	3.04	1.34 to 6.91	**0.008**	2.24	0.76 to 6–61	0.14	13.21	2.13 to 81.96	**0.006**
Years in school, < 13 years				0.36	0.13 to 0.96	**0.042**			
Ever smoker	1.26	0.61 to 2.58	0.53				1.35	0.38 to 4.76	0.64
VAS global fatigue, > median	6.36	3.06 to 13.19	**< 0.001**	7.73	2.63 to 22.76	**< 0.001**	17.29	3.82 to 78.23	**< 0.001**
Lateral spinal flexion, cm	1.02	0.95 to 1.09	0.64				0.94	0.81 to 1.10	0.44
BASFI, unit	1.18	0.93 to 1.50	0.16	1.41	0.99 to 2.00	0.057	1.07	0.73 to 1.57	0.72
ASDAS-CRP, ≥ 2.1	2.97	1.41 to 6.25	**0.004**	2.62	0.89 to 7.70	0.080	6.74	1.76 to 25.85	**0.005**
Log10mSASSS + 1, score							1.17	0.29 to 4.66	0.82
≥ 3 intervertebral bridges consecutively (only men)^a^				0.17	0.044 to 0.65	**0.01**			

Significant differences are in bold text. The independent variables were defined by having a univariate association with MCS (p-value ≤0.2) and not demonstrating collinearity. Due to some missing data, the models are based on overall 204 patients and in stratified analyses 117 men and 86 women

*MCS* mental component summary, *OR* odds ratio, *CI* confidence interval, *BMI* body mass index, *VAS* visual analogue scale, *BASFI* Bath Ankylosing Spondylitis Functional Index, *ASDAS-CRP* Ankylosing Spondylitis Disease Activity Score C-reactive protein, *mSASSS* Modified Stoke Ankylosing Spondylitis Spine Score, ^a^Cervical and/or lumbar spine

## Discussion

In this study, we have used the generic instrument SF-36 to evaluate HRQoL in a well-characterised cohort of patients with AS. The patients with AS had significantly lower HRQoL compared with controls. PCS was more affected compared to MCS in both sexes. Both disease-related and demographic factors were associated with HRQoL. Also, for the first time, to the best of our knowledge, we identified determinants of worse HRQoL separately in men and in women with AS and we found that the factors associated with HRQoL differed in part between the sexes.

We showed that the patients with AS had significantly lower SF-36 scores compared to 1055 age-matched and sex-matched controls from the general population. The patients with AS scored lower in all SF-36 domains and component summary scores. Our findings are in line with results from other countries in which SF-36 in AS has been compared indirectly with results from the general population [9, 22–24] and with the meta-analysis by Yang et al. who compared SF-36 scores in AS using two normative general-population databases of the SF-36. They stated also that HRQoL in AS was lower than in type II diabetes mellitus, and comparable with rheumatoid arthritis [7]. Thus, these results underline the importance of taking HRQoL into consideration in the management of patients with AS.

Furthermore, we observed that the PCS score was more affected compared to the MCS score. This was noted in the whole cohort and also in men and in women analysed individually. Yang et al. reported a pooled mean PCS score of 37.5 and MCS score of 44.7 [7]. Our results were similar, with a mean PCS score ± SD of 41.4 ± 10.0 and MCS score of 44.8 ± 12.5 (*n* = 210). This suggests that AS has a larger impact on physical components of HRQoL compared to mental components, although both dimensions are reduced compared to the general population.

It is known that the phenotype of AS differs between the sexes. For instance, at a group level, men with AS have more AS-related spinal radiographic changes [25]. This finding is one reason why it could be hypothesized that there should be more effect on HRQoL in men than in women with AS. However, we found that the women with AS scored worse in two mental domains and one physical domain compared to the men with AS. Similar sex differences in SF-36 scores have been reported previously [22, 26, 27]. Interestingly, results from the SF-36 Swedish normative population database, consisting of 8930 persons from the general population in different parts of Sweden, showed a clear difference in mean scores between men and women, with women generally reporting lower HRQoL. This indicates a general phenomenon that women report worse HRQoL compared to men [4].

We also explored the impact of age on the PCS and MCS scores. Concerning the MCS, the median MCS score was stable among the four different age groups in our study and was not correlated with age. However, the median PCS score was significantly worse in the age group with the oldest patients (≥ 61 years) compared to the group with the youngest patients (≤ 40 years). The pattern was similar in the SF-36 Swedish and Norwegian normative population databases displaying associations with age and the physical health domains but small differences for the mental domains across age groups [4, 28].

The multivariable logistic regression analysis in our study revealed that a patient was twice as likely to have a worse PCS score if they lived without a partner, and three times more likely to have a worse PCS score if they had ASDAS-CRP ≥ 2.1 vs ASDAS-CRP < 2.1. A longer symptom duration and higher BASFI were also associated with a worse PCS score. Analyses by sex revealed that a high BASFI and ASDAS-CRP were the most important determinants of a worse PCS score in the men, while in the women high BASFI/reduced physical function was associated with a worse PCS score. Salaffi et al. also found that high disease activity and chronic comorbidity were significantly associated with a lower PCS [9], and Özdemir reported inverse correlation between BASDAI, BASFI and most of the SF-36 domains [12]. We found that the pattern was somewhat different for the MCS score. A global fatigue score above the median value was associated with a six-fold increase in the risk of a worse MCS score. Patients living without a partner and patients who had an ASDAS-CRP ≥ 2.1 were three times more likely to have a worse MCS score. In analyses stratified by sex the same variables were significant in women, while in men, high levels of fatigue, longer education and absence of severe spinal radiographic changes, defined as ≥ 3 consecutive intervertebral bridges in the cervical spine and/or the lumbar spine were factors associated with worse MCS score. Opposite results on the level of education have previously been reported by some, with a low level of education or ≤ 12 years of education associated with worse HRQoL [9, 22, 26]. The discrepancy may have to do with our sex-stratified analyses and differences in selection and demographics between studies. We did not find the mSASSS for radiographic changes or the presence of syndesmophytes in the spine to be significant determinants of SF-36 scores, but severe spinal radiographic changes in men, defined as ≥ 3 consecutive intervertebral bridges in the cervical spine and/or the lumbar spine, were associated with better mental health, with MCS in multivariable analyses and with worse physical health, worse physical health, PCS in univariate but not in multivariable analyses. Importantly, it was only men in our study that had ≥ 3 consecutive intervertebral bridges in the cervical spine and/or the lumbar spine. Only a few previous studies have addressed if there is a relation between radiographic changes in the spine and HRQoL in AS. A Chinese study conducted in 245 patients with AS found that patients with severe kyphosis had a significantly worse PF domain score compared to patients with milder kyphosis [10]. In a study of 962 patients with AS, Bodur et al. identified weak but significant inverse correlation between the Bath Ankylosing Spondylitis Radiology Index (BASRI) and some of the SF-36 domains (PF, GH and MH) [29] similar to findings in a study of 100 patients with AS from Morocco [30]. Weak significant correlation was identified between the BASRI and worse Ankylosing Spondylitis Quality of Life questionnaire (ASQoL) scores in univariate but not in multivariate regression analyses in early axial SpA [31]. Conversely, a small study conducted in 36 patients with AS found no significant correlation between the mSASSS and SF-36 scores [32] and Machado et al. did not find significant association between the PCS or MCS scores and the mSASSS [13], while a higher mSASSS was significantly associated with better ASQoL in another study [33]. In a longitudinal study, the mSASSS at baseline was not associated with worse ASQoL over time [34]. In summary, previous results are not consistent, which can be explained by the use of different methods for grading spinal radiographic changes; the mSASSS, BASRI, kyphosis and ≥ 3 consecutive intervertebral bridges, different questionnaires assessing HRQoL; SF-36 and ASQoL, different statistical approaches; univariate and multivariable analyses and differences in methods of recruiting the patients etc. However, there seems to be some direct association between HRQoL and AS-related spinal radiographic changes. Interestingly, for the first time, to the best of our knowledge, we found that men who had severe spinal radiographic changes had better mental health compared to men without such changes and we hypothesize that it can be explained by adaptive coping strategies in patients with a longstanding chronic disease [35].

The impact on HRQoL of living alone and/or to be unmarried has previously been investigated in other diseases and in the general population, and to the best of our knowledge, for the first time in this study of patients with AS. The literature displays inconsistent results. In patients with chronic diseases such as cancer and multiple sclerosis, living alone is mostly associated with worse HRQoL, which is in line with the findings in our study [36–38]. However, in a longitudinal study, decline in the MCS score in patients with diabetes mellitus was associated with not living alone [39]. In a study from China, participants living alone had worse HRQoL [40], while the opposite was reported from another part of China, Shanghai [41]. Women in the Nurses’ Health Study who lived alone had lower risk of decline in the MH and VT domains compared with those living with a spouse. Furthermore, contact with friends and relatives and level of social engagement significantly protected against a decline in MH in women living alone but not among women living with a partner [42]. Thus, the influence of living alone on HRQoL is complex and seems to be related to the persons’ state of health, culture and gender, among other factors.

The sex-stratified analyses in our study revealed some differences; the disease activity seemed to be a more important factor in the physical component of HRQoL in men while the civil state was more important for the mental component of HRQoL in women, which are aspects that can be taken into account in the management of the patients with AS.

There are some limitations to be acknowledged; first this study is cross-sectional and thus we cannot draw any conclusions about the variables identified as associated with worse HRQoL and causality. To investigate this a longitudinal study is required. Second, the SF-36 Swedish normative population database was created in the 1990s and HRQoL in the general population might have changed over the years. However, HRQoL in the general population assessed by the SF-36 was stable from 1996 to 2004 in Norway [43], our neighbouring country, indicating that the difference in time may not be a significant problem. Third, we chose to divide the patients with AS according to their median values of PCS and MCS. Since there is no established cutoff for better or worse HRQoL we used this pragmatic approach. Furthermore, the modest number of subjects in the sex-stratified analyses result in broad confidence intervals and thus results need to be interpreted with some caution. Strengths of this study comprise first, a well-characterised patient cohort with an adequate number of patients allowing subgroup analyses of the sexes to be carried out for the first time. Second, SF-36 scores in patients with AS were compared to those in a large sample of controls from the general population who were precisely matched on sex and age.

Possible implications of our findings include that we have identified variables associated with worse HRQoL, which are modifiable. Even though our study is cross-sectional, and thus not able to show causality, by using these variables as a guide, patients may be treated more efficiently, leading to reduction in disease activity, pain and fatigue, thus potentially improving both the PCS and MCS scores as has been shown in some clinical trials [44–46]. To live alone without a partner was also associated with worse HRQoL in particular in women in the MCS score. A focus on social activities and community support for women with AS might help to improve MCS scores in this subset of patients with AS.

## Conclusions

In this study we show that the patients with AS had significantly lower HRQoL when assessed by the SF-36, compared with the general population. The PCS score was more affected than the MCS score in both sexes. Both demographic and disease-related factors were associated with worse HRQoL, with partial overlap for the PCS and MCS. There were some differences between sexes in the factors associated with HRQoL. By modifying factors such as ASDAS-CRP and fatigue, HRQoL may potentially be improved. There is a need for longitudinal studies investigating predictors related to the development of HRQoL in AS over time. We intend to investigate factors related to the change in HRQoL over a 5-year period in this same cohort of patients with AS in a future study.

## Additional files

Additional file 1: **Table S1.** Results of the SF-36 in patients with ankylosing spondylitis and in controls from the general population. **Table S2.** Comparisons of variables in patients with ankylosing spondylitis with scores below or above the median PCS score. **Table S3.** Comparisons of variables in patients with ankylosing and spondylitis scores below or above the median MCS score. **Table S4.** Univariate logistic regression analyses with PCS score below the median values as dependent variable and all assessed variables as covariates. **Table S5.** Univariate logistic regression analyses with MCS score below the median values as dependent variable and all assessed variables as covariates. **Table S6.** Multivariable logistic regression analyses with PCS and MCS scores below their median values as dependent variables and demographics as covariates. (DOCX 92 kb)

## Abbreviations

AS: Ankylosing spondylitis
ASAS: Assessment of SpondyloArthritis International Society
ASDAS: Ankylosing Spondylitis Disease Activity Score
BASDAI: Bath Ankylosing Spondylitis Disease Activity Index
BASFI: Bath Ankylosing Spondylitis Functional Index
BASG: Bath Ankylosing Spondylitis Patient Global
BASMI: Bath Ankylosing Spondylitis Metrology Index
BASRI: Bath Ankylosing Spondylitis Radiology Index
BMI: Body mass index
BP: Bodily pain
CI: Confidence interval
CRP: C-reactive protein
DMARD: Disease-modifying anti-rheumatic drug
ESR: Erythrocyte sedimentation rate
GH: General health
HLA-B27: Human leukocyte antigen B27
HRQoL: Health-related quality of life
IBD: Inflammatory bowel disease
MCS: Mental component summary
MH: Mental health
mSASSS: Modified Stoke Ankylosing Spondylitis Spine Score
NSAID: Non-steroid anti-inflammatory drug
OR: Odds ratio
PCS: Physical component summary
PF: Physical functioning
RE: Role emotional
RP: Role physical
SD: Standard deviation
SF: Social functioning
SF-36: Short form-36
TNFi: Tumor necrosis factor inhibitor
VAS: Visual analogue scale
VT: Vitality
