# Supplementary material for: Factors related to health-related quality of life in ankylosing spondylitis, overall and stratified by sex
Source: Arthritis Res Ther. 2018 Dec 27;20:284. doi: 10.1186/s13075-018-1784-8 (PMC6307231; doi:10.1186/s13075-018-1784-8)
Supplement: Supplementary file 1 — Table S1. Results of the SF-36 in patients with ankylosing spondylitis and in controls from the general population. Table S2. Comparisons of variables in patients with ankylosing spondylitis with scores below or above the median PCS score. Table S3. Comparisons of variables in patients with ankylosing and spondylitis scores below or above the median MCS score. Table S4. Univariate logistic regression analyses with PCS score below the median values as dependent variable and all assessed variables as covariates. Table S5. Univariate logistic regression analyses with MCS score below the median values as dependent variable and all assessed variables as covariates. Table S6. Multivariable logistic regression analyses with PCS and MCS scores below their median values as dependent variables and demographics as covariates. (DOCX 92 kb) [file 13075_2018_1784_MOESM1_ESM.docx]

**Additional files**

Table S1. Results of SF-36 in patients with ankylosing spondylitis and in controls from the general population

| **SF-36 Domain** | **Statistics** | **Controls (n=1055)** | **AS patients (n=211)** | **P value** |
| --- | --- | --- | --- | --- |
| PF | Median (IQR) | 95.0 (20.0) | 80.0 (25.0) | **<0.001** |
|  | Mean ± SD | 86.6 ± 19.5 | 74.5 ± 21.2 |  |
| RP | Median (IQR) | 100.0 (25.0) | 75.0 (75.0) | **<0.001** |
|  | Mean ± SD | 81.7 ± 32.7 | 59.4 ± 41.2 |  |
| BP | Median (IQR) | 84.0 (49.0) | 51.0 (23.0) | **<0.001** |
|  | Mean ± SD | 72.9 ± 27.0 | 53.9 ± 22.2 |  |
| GH | Median (IQR) | 80.0 (30.0) | 62.0 (37.0) | **<0.001** |
|  | Mean ± SD | 74.1 ± 22.8 | 57.2 ± 22.9 |  |
| VT | Median (IQR) | 75.0 (30.0) | 50.0 (40.0) | **<0.001** |
|  | Mean ± SD | 69.8 ± 23.2 | 50.4 ± 23.9 |  |
| SF | Median (IQR) | 100.0 (12.5) | 75.0 (37.5) | **<0.001** |
|  | Mean ± SD | 88.1 ± 21.2 | 75.4 ± 25.6 |  |
| RE | Median (IQR) | 100.0 (0.00) | 100.0 (66.7) | **<0.001** |
|  | Mean ± SD | 86.4 ± 28.2 | 68.4 ± 41.0 |  |
| MH | Median (IQR) | 88.0 (24.0) | 76.0 (28.0) | **<0.001** |
|  | Mean ± SD | 81.2 ± 19.6 | 71.5 ± 20.1 |  |
| PCS | Median (IQR) | 52.4 (11.8) | 42.4 (14.5) | **<0.001** |
|  | Mean ± SD | 49.1 ± 9.8 | 41.3 ± 10.1 |  |
| MCS | Median (IQR) | 54.1 (10.1) | 47.9 (20.0) | **<0.001** |
|  | Mean ± SD | 50.4 ± 10.4 | 44.6 ±12.7 |  |

Significant differences are in bold text. Comparisons are assessed by Mann-Whitney U test.

PCS; physical component summary, MCS; mental component summary, AS; ankylosing spondylitis, IQR; interquartile range, SD; standard deviation PF; physical function, RP; role physical, BP; body pain, GH; general health, VT; vitality, SF; social function, RE; role emotional, MH; mental health,

Table S2. Comparisons of variables in ankylosing spondylitis patients with score below or above the median PCS score.

|  | All, n=210 | | | Men, n=121 | | | Women, n=89 | | |
| --- | --- | --- | --- | --- | --- | --- | --- | --- | --- |
|  | **PCS< 42.4** | **PCS> 42.4** | **P-value** | **PCS< 43.6** | **PCS> 43.6** | **P-value** | **PCS< 41.8** | **PCS> 41.8** | **P-value** |
| Women, n (%) | 46 (44.2) | 42 (40.4) | 0.58 |  |  |  |  |  |  |
| Age, years | 52.7 (43.0, 64.0) | 47.5 (38.0, 56.8) | **0.001** | 52.5 (41.5, 64.0) | 48.0 (35.2, 57.0) | **0.02** | 54.0 (44.2, 64.0) | 47.0 (39.5, 56.5) | **0.03** |
| BMI, kg/m^2^ | 25.8 (23.2, 29.0) | 24.5 (22.7, 27.3) | 0.061 | 26.1 (23.8, 29.6) | 25.0 (23.4, 28.2) | 0.13 | 24.4 (22.5, 28.0) | 23.7 (21.2, 26.6) | 0.21 |
| Civil state, single, n (%) | 36 (64.3) | 20 (35.7) | **0.012** | 21 (63.6) | 12 (36.4) | 0.066 | 16 (69.6) | 7 (30.4) | **0.029** |
| Years in school, < 13 years, n (%) | 54 (56.3) | 42 (43.8) | 0.11 | 34 (55.7) | 27 (44.3) | 0.17 | 20 (57.1) | 15 (42.9) | 0.28 |
| Ever smoker, n (%) | 58 (55.8) | 47 (45.2) | 0.12 | 37 (61.7) | 28 (46.7) | 0.099 | 21 (47.7) | 19 (43.2) | 0.67 |
| VAS global fatigue, score | 65.0 (50.0, 79.0) | 35.0 (15.0, 68.0) | **<0.001** | 65.0 (46.2, 79.0) | 23.5 (11.0, 56.5) | **<0.001** | 65.0 (50.0, 80.0) | 61.5 (25.2, 75.0) | 0.098 |
| Duration of symptoms, years | 27.0 (16.0, 38.0) | 18.5 (11.0, 18.0) | **<0.001** | 26.5 (17.2, 37.0) | 17.0 (11.0, 28.0) | **0.001** | 27.0 (15.0, 39.0) | 21.5 (11.0, 30.8) | 0.091 |
| ESR, mm/h | 12.0 (8.0, 24.0) | 10.5 (6.0, 17.0) | **0.019** | 11.0 (7.0, 23.2) | 9.0 (5.0, 14.5) | 0.14 | 15.0 (11.0, 24.8) | 11.5 (7.0, 20.8) | 0.073 |
| CRP, mg/L | 3.0 (1.0, 8.0) | 2.0 (1.0, 5.75) | 0.094 | 3.0 (2.0, 8.0) | 2.5 (1.0, 6.0) | 0.31 | 3.0 (1.0, 8.0) | 2.0 (0.0, 4.0) | 0.065 |
| HLA-B27, n (%) | 89 (85.6) | 92 (88.5) | 0.54 | 56 (93.3) | 55 (91.7)) | 0.73 | 34 (77.3) | 36 (81.8)) | 0.60 |
| History of anterior uveitis, n (%) | 52 (50.0) | 54 (51.9) | 0.78 | 33 (55.0) | 38 (58.3) | 0.71 | 20 (45.5) | 18 (40.9) | 0.68 |
| TNFi and/or DMARD, n (%) | 35 (33.7) | 38 (36.5) | 0.66 | 22 (36.7) | 23 (38.3) | 0.85 | 13 (29.5) | 15 (34.1) | 0.65 |
| BASMI, score | 3.3 (2.2, 4.6) | 2.5 (1.6, 3.6) | **<0.001** | 3.4 (2.2, 5.6) | 2.5 (1.4, 3.6) | **0.001** | 3.2 (2.4, 4.2) | 2.5 (1.8, 3.0) | **0.003** |
| Lateral spinal flexion, cm | 11.5 (7.5, 15.4) | 14.8 (11.0, 19.0) | **<0.001** | 11.4 (5.2, 15.9) | 15.0 (11.1, 20.4) | **0.001** | 11.5 (7.8, 14.5) | 13.6 (10.6, 18.9) | **0.022** |
| Modified Schober, cm | 4.0 (3.0, 4.5) | 4.0 (3.1, 5.0 | **0.023** | 3.5 (2.0, 4.0) | 4.0 (3.0, 5.0 | **0.005** | 4.0 (3.0, 4.5) | 4.0 (3.6, 4.9 | 0.31 |
| BASFI, score | 3.5 (2.2, 5.4) | 1.1 (0.5, 2.4) | **<0.001** | 3.4 (2.3, 5.4) | 1.0 (0.5, 2.3) | **<0.001** | 3.7 (2.1, 5.3) | 1.1 (0.6, 2.4) | **<0.001** |
| BASDAI, score | 5.0 (3.1, 6.1) | 2.2 (1.1, 3.6) | **<0.001** | 5.0 (3.5, 6.1) | 1.7 (0.88, 2.7) | **<0.001** | 4.8 (2.9, 6.1) | 3.0 (1.6, 4.5) | **0.001** |
| ASDAS-CRP, score | 2.7 (1.9, 3.3) | 1.6 (1.2, 2.2) | **<0.001** | 2.8 (2.1, 3.2) | 1.5 (1.1, 2.0) | **<0.001** | 2.6 (1.8, 3.3) | 1.9 (1.3, 2.3) | **<0.001** |
| BAS-G, score | 4.8 (2.2, 6.6) | 1.4 (0.7, 3.5) | **<0.001** | 4.7 (2.4, 6.6) | 1.2 (0.4, 2.7) | **<0.001** | 4.9 (2.1, 6.8) | 1.6 (0.9, 3.7) | **<0.001** |
| mSASSS, score | 6.0 (0.0, 20.0) | 4.0 (0.0, 18.8) | 0.18 | 11.5 (4.0, 49.0) | 7.5 (2.0, 25.8) | 0.094 | 3.5 (0.0, 11.8) | 0.5 (0.0, 6.0) | 0.26 |
| ≥ Syndesmophyte, n (%) | 51 (52.0) | 47 (48.0) | 0.58 | 39 (65.0) | 32 (53.3) | 0.19 | 13 (29.5) | 14 (31.8) | 0.82 |
| ≥3 consecutive inter-vertebral bridges (only men)# |  |  |  | 20 (69.0) | 9 (31.0) | **0.019** |  |  |  |
| MCS, score | 44.2 (31.1, 52.9) | 51.3 (41.7, 56.2) | **0.003** | 44.6 (28.8, 53.2) | 52.3 (46.8, 56.6) | **0.001** | 44.6 (33.5, 53.9) | 48.6 (33.1, 53.8) | 0.87 |
| PCS, score | 34.3 (28.4, 38.5) | 48.7 (46.1, 53.1) | **<0.001** | 35.5 (28.5, 40.1) | 49.3 (47.1, 53.2) | **<0.001** | 33.0 (26.9, 36.4) | 47.5 (43.9, 52.6) | **<0.001** |

Values are median and 25^th^ percentile (Q1) and 75^th^ percentile (Q3) or numbers of patients and percent (%). Comparisons are assessed by Mann-Whitney U test or Chi square test. Significant differences are in bold text.
PCS; physical component summary, BMI; Body Mass Index, VAS; Visual Analogue Scale, ESR; erythrocyte sedimentation rate, CRP; C-reactive protein, TNFi; TNF inhibitor, csDMARD; conventional synthetic disease modifying anti-rheumatic drug, BASMI; Bath Ankylosing Spondylitis Metrology Index, BASFI; Bath Ankylosing Spondylitis Functional Index, BASDAI; Bath Ankylosing Spondylitis Disease Activity Index , ASDAS-CRP; Ankylosing Spondylitis Disease Activity Score CRP, BAS-G; Bath Ankylosing Spondylitis Patient Global, mSASSS; Modified Stoke Ankylosing Spondylitis Spine Score, MCS; mental component summary, #cervical and/or lumbar spine

Table S3. Comparisons of variables in ankylosing spondylitis patients with score below or above the median MCS score.

|  | All, n=210 | | | Men, n=121 | | | Women, n=89 | | |
| --- | --- | --- | --- | --- | --- | --- | --- | --- | --- |
|  | **MCS< 48.0** | **MCS> 48.0** | **P-value** | **MCS< 49.2** | **MCS> 49.2** | **P-value** | **MCS< 46.0** | **MCS> 46.0** | **P-value** |
| Female sex, n (%) | 48 (46.2) | 40 (38.5) | 0.26 |  |  |  |  |  |  |
| Age, years | 49.0 (40.2, 61.8) | 50.0 (39.2, 61.0) | 0.79 | 49.0 (39.2, 60.8) | 49.5 (38.5, 61.8) | 0.77 | 49.0 (42.2, 61.2) | 52.0 (41.0, 62.0) | 0.65 |
| BMI, kg/m^2^ | 26.4 (22.7, 29.0) | 24.7 (23.0, 27.6) | 0.58 | 25.8 (23.5, 29.9) | 25.6 (23.6, 28.6) | 0.63 | 23.9 (21.7, 27.6) | 24.2 (21.8, 27.2) | 0.97 |
| Civil state, single, n (%) | 36 (64.3) | 20 (35.7) | **0.012** | 20 (60.6) | 13 (39.4) | 0.15 | 17 (74.9) | 6 (26.1) | **0.008** |
| Years in school, < 13 years, n (%) | 45 (46.9) | 51 (53.1) | 0.37 | 27 (44.3) | 34 (55.7) | 0.17 | 17 (48.6) | 18 (51.4) | 0.83 |
| Ever smoker, n (%) | 58 (55.8) | 47 (45.2) | 0.13 | 34 (56.7) | 31 (51.7) | 0.58 | 24 (54.5) | 16 (36.4) | 0.087 |
| VAS global fatigue, score | 65.5 (58.2, 80.0) | 30.5 (15.2, 59.8) | **<0.001** | 65.0 (52.8, 78.2) | 27.0 (11.2, 50.0) | **<0.001** | 74.5 (60.8, 83.0) | 50 (26.2, 64.8) | **<0.001** |
| Duration of symptoms, years | 24.0 (14.0, 37.0) | 23.0 (11.0, 31.0) | 0.96 | 23.0 (14.0, 36.0) | 22.0 (11.0, 31.0) | 0.23 | 24.0 (14.0, 37.8) | 24.0 (12.0, 32.0) | 0.66 |
| ESR, mm/h | 11.0 (7.0, 20.0) | 12.0 (7.0, 18.8) | 0.90 | 10.0 (5.0, 15.2) | 10.5 (7.0, 17.8) | 0.26 | 13.0 (9.0, 23.8) | 14.0 (10.2, 20.8) | 0.70 |
| CRP, mg/L | 2.0 (1.0, 7.8) | 3.0 (1.0, 7.0) | 0.86 | 3.0 (0.2, 6.8) | 3.0 (1.2, 7.0) | 0.78 | 2.0 (1.0, 8.0) | 2.0 (0.0, 5.0) | 0.42 |
| HLA-B27, n (%) | 89 (85.6) | 92 (88.5) | 0.54 | 55 (91.7) | 56 (93.3) | 0.73 | 35 (79.5) | 35 (79.5) | 1.00 |
| History of anterior uveitis, n (%) | 51 (49.0) | 55 (52.9) | 0.58 | 31 (51.7) | 37 (61.7) | 0.27 | 22 (50.0) | 16 (36.4) | 0.20 |
| TNFi and/or csDMARD, n (%) | 34 (32.7) | 39 (37.5) | 0.47 | 22 (36.7) | 23 (38.3) | 0.85 | 12 (27.3) | 16 (36.4) | 0.36 |
| BASMI, score | 3.0 (2.0, 4.0) | 2.8 (1.8, 4.0) | 0.36 | 2.9 (1.8, 4.0) | 3.2 (1.8, 4.8) | 0.70 | 3.0 (2.2, 4.2) | 2.6 (1.8, 3.4) | 0.069 |
| Lateral spinal flexion, cm | 11.9 (9.0, 16.4) | 13.5 (9.6, 18.2) | 0.11 | 12.9 (9.1, 18.0) | 13.5 (8.0, 17.2) | 0.77 | 11.4 (8.1, 14.5) | 13.5 (10.2, 18.5) | **0.016** |
| Modified Schober, cm | 4.0 (3.0, 4.5) | 4.0 (3.0, 5.0) | 0.62 | 4.0 (3.0, 4.4) | 4.0 (2.1, 4.9) | 0.92 | 4.0 (3.5, 4.5) | 4.0 (3.5, 4.5) | 0.98 |
| BASFI, score | 3.2 (1.6, 4.9) | 1.6 (0.7, 2.8) | **<0.001** | 3.1 (1.5, 5.1) | 1.6 (0.7, 2.8) | **<0.001** | 3.6 (1.8, 5.1) | 1.6 (0.8, 2.9) | **<0.001** |
| BASDAI, score | 4.8 (3.1, 6.0) | 2.0 (1.4, 3.5) | **<0.001** | 4.4 (2.5, 5.9) | 1.8 (1.1, 3.3) | **<0.001** | 5.1 (3.8, 6.1) | 2.4 (1.6, 3.6) | **<0.001** |
| ASDAS-CRP, score | 2.6 (1.9, 3.2) | 1.7 (1.2, 2.2) | **<0.001** | 2.8 (1.8, 3.2) | 1.6 (1.3, 2.1) | **<0.001** | 2.6 (2.0, 3.3) | 1.7 (1.1, 2.2) | **<0.001** |
| BAS-G, score | 4.8 (2.8, 6.6) | 1.4 (0.7, 3.0) | **<0.001** | 4.6 (2.2, 6.6) | 1.6 (0.7, 2.9) | **<0.001** | 5.0 (3.5, 6.6) | 1.4 (0.8, 3.3) | **<0.001** |
| mSASSS, score | 5.0 (0.0, 19.0) | 6.0 (0.0, 24.2) | 0.48 | 7.5 (2.0, 22.2) | 16.5 (2.0, 39.0) | 0.25 | 2.5 (0.0, 12.2) | 0.5 (0.0, 6.0) | 0.205 |
| ≥ Syndesmophyte, n (%) | 47 (48) | 51 (52) | 0.58 | 37 (61.7) | 34 (56.7) | 0.58 | 11 (25.0) | 16 (36.4) | 0.25 |
| ≥3 consecutive inter-vertebral bridge (only men)# |  |  |  | 11 (37.9) | 18 (62.1) | 0.14 |  |  |  |
| MCS, score | 35.7 (27.2, 43.9) | 55.1 (51.6, 57.9) | **<0.001** | 40.0 (27.2, 45.3) | 56.0 (52.6, 58.3) | **<0.001** | 33.6 (26.8, 40.6) | 53.7 (50.1, 57.7) | **<0.001** |
| PCS, score | 39.4 (31.5, 46.3) | 45.9 (36.8, 50.3) | **<0.002** | 39.7 (31.9, 46.9) | 47.2 (40.1, 50.7) | **0.001** | 38.9 (30.6, 46.0) | 43.4 (34.6, 49.0) | 0.16 |

Values are median and 25^th^ percentile (Q1) and 75^th^ percentile (Q3) or numbers of patients and percent (%). Comparisons are assessed by Mann-Whitney U test or Chi square test. Significant differences are in bold text.
MCS; mental component summary BMI; Body Mass Index, VAS; Visual Analogue Scale, ESR; erythrocyte sedimentation rate, CRP; C-reactive protein, TNFi; TNF inhibitor, csDMARD; conventional synthetic disease modifying anti-rheumatic drug, BASMI; Bath Ankylosing Spondylitis Metrology Index, BASFI; Bath Ankylosing Spondylitis Functional Index, BASDAI; Bath Ankylosing Spondylitis Disease Activity Index , ASDAS-CRP; Ankylosing Spondylitis Disease Activity Score CRP, BAS-G; Bath Ankylosing Spondylitis Patient Global, mSASSS; Modified Stoke Ankylosing Spondylitis Spine Score, PCS; physical component summary, #cervical and/or lumbar spine

Table S4.Univariate logistic regression analyses with PCS score below the median values as dependent variable and all assessed variables as covariates.

|  | All, n=210  PCS < median PCS 42.4 | | | Men, n=121 PCS < median PCS 43.6 | | | Women, n=89 PCS < median PCS 41.8 | | |
| --- | --- | --- | --- | --- | --- | --- | --- | --- | --- |
| Variables | **OR** | **95% CI** | ***P*-value** | **OR** | **95% CI** | ***P*-value** | **OR** | **95% CI** | ***P*-value** |
| Age, decades | 1.40 | 1.13 to 1.74 | **0.002** | 1.40 | 1.06 to 1.85 | **0.017** | 1.36 | 0.96 to 1.91 | 0.082 |
| BMI, kg/m^2^ | 1.07 | 1.01 to 1.15 | **0.029** | 1.09 | 1.00 to 1.19 | 0.060 | 1.06 | 0.96 to 1.16 | 0.26 |
| Civil state, single | 2.22 | 1.18 to 4.19 | **0.013** | 2.15 | 0.94 to 4.92 | 0.069 | 3.02 | 1.10 to 8.33 | **0.033** |
| Years in school, < 13 years | 1.57 | 0.90 to 2.72 | 0.11 | 1.66 | 0.80 to 3.43 | 0.17 | 1.61 | 0.68 to 3.81 | 0.28 |
| Ever smoker | 1.53 | 0.88 to 2.64 | 0.13 | 1.84 | 0.89 to 3.80 | 0.10 | 1.20 | 0.52 to 2.78 | 0.67 |
| VAS global fatigue, > median | 2.78 | 1.58 to 4.87 | **<0.001** | 6.10 | 2.74 to 13.58 | **<0.001** | 1.10 | 0.47 to 2.56 | 0.83 |
| Duration of symptoms, decades | 1.72 | 1.33 to 2.23 | **<0.001** | 1.85 | 1.29 to 2.64 | **<0.001** | 1.49 | 1.02 to 2.14 | **0.038** |
| ESR, > median | 1.59 | 0.92 to 2.75 | 0.097 | 1.32 | 0.64 to 2.75 | 0.46 | 2.14 | 0.90 to 5.10 | 0.085 |
| CRP, > median | 1.46 | 0.84 to 2.53 | 0.18 | 1.50 | 0.73 to 3.11 | 0.27 | 1.68 | 0.71 to 3.99 | 0.24 |
| HLA-B27 | 0.77 | 0.34 to 1.74 | 0.54 | 1.27 | 0.32 to 4.99 | 0.73 | 0.76 | 0.27 to 2.14 | 0.60 |
| History of anterior uveitis | 0.93 | 0.54 to 1.60 | 0.78 | 0.87 | 0.42 to 1.80 | 0.71 | 1.20 | 0.52 to 2.80 | 0.67 |
| TNFi and/or csDMARD | 0.88 | 0.50 to 1.56 | 0.66 | 0.93 | 0.44 to 1.95 | 0.85 | 0.81 | 0.33 to 1.99 | 0.65 |
| BASMI, unit | 1.51 | 1.24 to 1.84 | **<0.001** | 1.48 | 1.18 to 1.86 | **<0.001** | 1.76 | 1.16 to 2.67 | **0.007** |
| Lateral spinal flexion, cm | 0.90 | 0.85 to 0.95 | **<0.001** | 0.89 | 0.83 to 0.95 | **<0.001** | 0.89 | 0.82 to 0.98 | **0.016** |
| Modified Schober, cm | 0.76 | 0.62 to 0.94 | **0.013** | 0.67 | 0.51 to 0.87 | **0.003** | 0.81 | 0.55 to 1.20 | 0.30 |
| BASFI, unit | 2.24 | 1.77 to 2.83 | **<0.001** | 2.64 | 1.85 to 3.79 | **<0.001** | 2.19 | 1.55 to 3.11 | **<0.001** |
| BASDAI ≥ 4 | 6.85 | 3.64 to 12.91 | **<0.001** | 22.0 | 7.56 to 64.03 | **<0.001** | 2.98 | 1.24 to 7.16 | **0.015** |
| ASDAS-CRP, ≥ 2.1 | 6.29 | 3.44 to 11.51 | **<0.001** | 10.56 | 4.53 to 24.64 | **<0.001** | 3.38 | 1.41 to 8.18 | **0.006** |
| BAS-G, > median | 5.81 | 3.20 to 10.55 | **<0.001** | 9.33 | 4.03 to 21.61 | **<0.001** | 3.44 | 1.42 to 8.33 | **0.006** |
| Log10mSASSS+1, score | 1.37 | 0.88 to 2.12 | 0.16 | 1.58 | 0.86 to 2.87 | 0.14 | 1.62 | 0.74 to 3.55 | 0.23 |
| ≥ Syndesmophyte | 1.17 | 0.68 to 2.01 | 0.58 | 1.62 | 0.78 to 3.38 | 0.20 | 0.90 | 0.36 to 2.22 | 0.82 |
| ≥3 consecutive inter-vertebral bridges (only men)# |  |  |  | 2.83 | 1.16 to 6.89 | **0.022** |  |  |  |

Significant differences are in bold text. PCS; physical component summary, OR; odds ratio, CI; confidence interval, BMI; Body Mass Index, VAS; Visual Analogue Scale, ESR; erythrocyte sedimentation rate, CRP; C-reactive protein, TNFi; TNF inhibitor, csDMARD; conventional synthetic disease modifying anti-rheumatic drug, BASMI; Bath Ankylosing Spondylitis Metrology Index, BASFI; Bath Ankylosing Spondylitis Functional Index, BASDAI; Bath Ankylosing Spondylitis Disease Activity Index, ASDAS-CRP; Ankylosing Spondylitis Disease Activity Score CRP, BAS-G; Bath Ankylosing Spondylitis Patient Global, mSASSS; Modified Stoke Ankylosing Spondylitis Spine Score, #cervical and/or lumbar spine

Table S5. Univariate logistic regression analyses with MCS score below the median values as dependent variable and all assessed variables as covariates.

|  | All, n=210  MCS < median MCS 48.0 | | | Men, n=121 MCS < median MCS 49.2 | | | Women, n=89 MCS < median MCS 46.0 | | |
| --- | --- | --- | --- | --- | --- | --- | --- | --- | --- |
| Variables | **OR** | **95% CI** | ***P*-value** | **OR** | **95% CI** | ***P*-value** | **OR** | **95% CI** | ***P*-value** |
| Age, decades | 1.00 | 0.82 to 1.24 | 0.96 | 1.02 | 0.78 to 1.32 | 0.89 | 0.93 | 0.67 to 1.30 | 0.68 |
| BMI, kg/m^2^ | 1.04 | 0.98 to 1.11 | 0.20 | 1.04 | 0.95 to 1.13 | 0.39 | 1.03 | 0.94 to 1.14 | 0.49 |
| Civil state, single | 2.22 | 1.18 to 4.19 | **0.013** | 1.81 | 0.80 to 4.09 | 0.16 | 3.99 | 1.39 to 11.43 | **0.010** |
| Years in school, < 13 years | 0.78 | 0.45 to 1.34 | 0.37 | 0.60 | 0.29 to 1.24 | 0.17 | 0.91 | 0.39 to 2.14 | 0.83 |
| Ever smoker | 1.53 | 0.88 to 2.64 | 0.13 | 1.22 | 0.60 to 2.51 | 0.58 | 2.10 | 0.89 to 4.93 | 0.089 |
| VAS global fatigue, > median | 8.13 | 4.38 to 15.12 | **<0.001** | 10.4 | 4.42 to 24.46 | **<0.001** | 8.70 | 3.24 to 23.36 | **<0.001** |
| Duration of symptoms, decades | 1.08 | 0.86 to 1.34 | 0.49 | 1.13 | 0.83 to 1.53 | 0.44 | 1.10 | 0.78 to 1.56 | 0.60 |
| ESR, > median | 0.79 | 0.46 to 1.37 | 0.40 | 0.57 | 0.27 to 1.20 | 0.14 | 0.69 | 0.29 to 1.61 | 0.39 |
| CRP, > median | 1.11 | 0.64 to 1.92 | 0.72 | 1.31 | 0.64 to 2.71 | 0.46 | 1.28 | 0.54 to 3.03 | 0.57 |
| HLA-B27 | 0.77 | 0.34 to 1.74 | 0.54 | 0.79 | 0.20 to 3.08 | 0.73 | 1.00 | 0.36 to 2.82 | 1.00 |
| History of anterior uveitis | 0.86 | 0.50 to 1.48 | 0.58 | 0.66 | 0.32 to 1.37 | 0.27 | 1.75 | 0.75 to 4.10 | 0.20 |
| TNFi and/or csDMARD | 0.81 | 0.46 to 1.43 | 0.47 | 0.93 | 0.44 to 1.95 | 0.85 | 0.66 | 0.27 to 1.62 | 0.36 |
| BASMI, unit | 1.05 | 0.89 to 1.25 | 0.54 | 0.96 | 0.79 to 1.17 | 0.69 | 1.49 | 1.01 to 2.20 | **0.045** |
| Lateral spinal flexion, cm | 0.96 | 0.92 to 1.01 | 0.15 | 0.99 | 0.93 to 1.05 | 0.75 | 0.89 | 0.82 to 0.98 | **0.016** |
| Modified Schober, cm | 0.97 | 0.79 to 1.18 | 0.74 | 1.04 | 0.82 to 1.33 | 0.73 | 0.84 | 0.57 to 1.24 | 0.39 |
| BASFI, unit | 1.54 | 1.30 to 1.82 | **<0.001** | 1.53 | 1.22 to 1.91 | **<0.001** | 1.58 | 1.22 to 2.05 | **0.001** |
| BASDAI ≥ 4 | 7.62 | 4.01 to 14.48 | **<0.001** | 5.70 | 2.47 to 13.17 | **<0.001** | 15.38 | 5.33 to 44.358 | **<0.001** |
| ASDAS-CRP, ≥ 2.1 | 5.73 | 3.15 to 10.42 | **<0.001** | 6.25 | 2.82 to 13.84 | **<0.001** | 6.36 | 2.52 to 16.07 | **<0.001** |
| BAS-G, > median | 8.57 | 4.58 to 15.96 | **<0.001** | 8.57 | 4.58 to 15.96 | **<0.001** | 10.73 | 3.94 to 29.26 | **<0.001** |
| Log10mSASSS+1, score | 0.84 | 0.55 to 1.31 | 0.45 | 0.73 | 0.40 to 1.32 | 0.30 | 1.67 | 0.76 to 3.66 | 0.20 |
| ≥ Syndesmophyte | 0.86 | 0.50 to 1.49 | 0.58 | 1.23 | 0.59 to 2.55 | 0.58 | 0.58 | 0.23 to 1.46 | 0.25 |
| ≥3 consecutive inter-vertebral bridges (only men)# |  |  |  | 0.52 | 0.22 to 1.23 | 0.14 |  |  |  |

Significant differences are in bold text. MCS; mental component summary, OR; odds ratio, CI; confidence interval, BMI; Body Mass Index, VAS; Visual Analogue Scale, ESR; erythrocyte sedimentation rate, CRP; C-reactive protein, TNFi; TNF inhibitor, csDMARD; conventional synthetic disease modifying anti-rheumatic drug, BASMI; Bath Ankylosing Spondylitis Metrology Index, BASFI; Bath Ankylosing Spondylitis Functional Index, BASDAI; Bath Ankylosing Spondylitis Disease Activity Index, ASDAS-CRP; Ankylosing Spondylitis Disease Activity Score CRP, BAS-G; Bath Ankylosing Spondylitis Patient Global, mSASSS; Modified Stoke Ankylosing Spondylitis Spine Score, #cervical and/or lumbar spine

Table S6. Multivariable logistic regression analyses with PCS and MCS scores below their median values as dependent variables and demographics as covariates.

|  | All, n=210  PCS < median PCS 42.4 | | | All, n=210  MCS < median MCS 48.0 | | |
| --- | --- | --- | --- | --- | --- | --- |
| Variables | **OR** | **95% CI** | ***P*-value** | **OR** | **95% CI** | ***P*-value** |
| BMI, kg/m^2^ | 1.05 | 0.98 to 1.13 | 0.14 | 1.05 | 0.98 to 1.12 | 0.14 |
| Civil state, single | 2.52 | 1.28 to 4.96 | **0.007** | 2.25 | 1.18 to 4.29 | **0.014** |
| Years in school, < 13 years | 1.38 | 0.75 to 2.54 | 0.30 |  |  |  |
| Ever smoker | 1.08 | 0.59 to 1.98 | 0.81 | 1.45 | 0.81 to 2.59 | 0.21 |
| Age, decades | 1.32 | 1.04 to 1.67 | **0.020** | 0.92 | 0.74 to 1.16 | 0.50 |

Significant differences are in bold text. PCS; physical component summary, MCS; mental component summary, OR; odds ratio, CI; confidence interval, BMI; Body Mass Index, The independent variables were defined by having a univariate association with PCS or MCS (p-value ≤ 0.2) and not demonstrating collinearity. In addition age, decades was added as a covariate for both PCS and MCS. Due to some missing data, the models are based on 208 patients concerning both PCS and MCS.
